# A patient with 18p11.32-p11.21 deletion have monaural deafness caused by an inadequate haplodose of THOC1: A case report

**DOI:** 10.1097/MD.0000000000039048

**Published:** 2024-07-26

**Authors:** Geng Ouyang, Enhuan Yi, Huali Qin, Xingxing Duan, Sifeng Wang, Xiangwen Peng

**Affiliations:** a Central Laboratory, Hunan Provincial Key Laboratory of Regional Hereditary Birth Defects Prevention and Control, Changsha Hospital for Maternal & Child Health Care Affiliated to Hunan Normal University, Changsha, China; b Central Laboratory, Changsha Hospital for Maternal and Child Health Care Affiliated to Hunan Normal University, Changsha, China.

**Keywords:** THOC1, unilateral deafness

## Abstract

**Background:**

THOC1 mutation causes Deafness, autosomal dominant 86 [OMIM: 620280]. However, it has not been reported whether deletion of the THOC1 gene causes deafness.

**Methods:**

Here, we report a 1-year-old girl with clinical features including Hypotonia, unilateral deafness in the right ear, and widening of lateral ventricles in 6 months. Gene mutations were identified by whole-exome sequencing.

**Results:**

Through whole-exome sequencing, a deletion of 18p11.32-p11.21 contains the deletion of all THOC1 genes found in the patient but not in her parents’ genomic DNA. The ClinGen Database Haplodose Insufficiency (HI) prediction tool determined that HI, THOC1 HI may cause unilateral deafness. Moreover, after 6 months of rehabilitation training, muscle tone returned to normal. However, at the age of 1 year, the patient developed symptoms of a large liver and hamartoma of both kidneys.

**Conclusion:**

From the above results, we propose that in our patient, THOC1 HI may cause unilateral deafness. Therefore, this study provides a new THOC1 deletion associated with unilateral deafness.

## 1. Introduction

Inherited forms of deafness are highly prevalent and severe conditions that significantly affect 5% of the world’s population, the lack of therapeutic options for these conditions poses a major socioeconomic burden.^[1,2]^ So far, many genetic factors have been found to cause deafness. However, there is a lack of treatments to prevent hearing loss due to genetic causes.^[2]^ The discovery of new genetic pathogenic genes is particularly important for the prevention and treatment of deafness. A recent study found that THOC1 deficiency leads to late-onset nonsyndromic hearing loss in zebrafish,^[3]^ but no case of deletion in THOC1 has been reported. It has been show that THOC1 affected mouse development. Loss of Thoc1 causes periimplantation embryonic lethality in the mouse. Knockdown of Thoc1 during mouse development showing a dwarf phenotype, suggesting that Thoc1 is also required during late embryonic and postnatal development.^[4,5]^ But the function of Thoc1 deficiency after birth in humans is still unclear. In our study, we reported the deletion in THOC1 gene may cause unilateral right ear deafness by expressing insufficient dosage of THOC1.

## 2. Results

**Figure 1.**
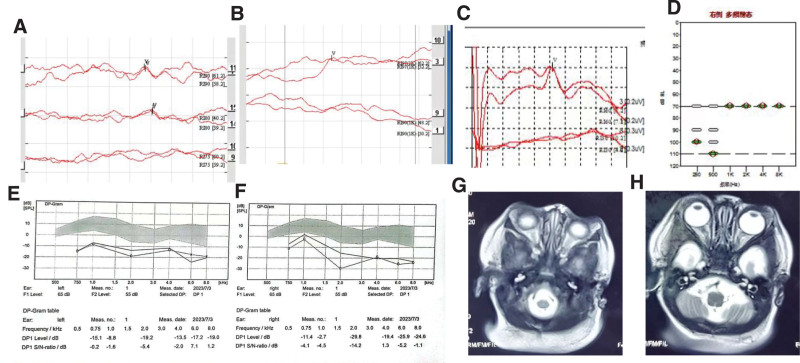
No organic abnormalities in the ear but hearing loss in the right ear. (A) Right ear bone conduction ABR threshold is 60dBHL. (B) right ear multi-frequency steady-state auditory evoked potential (ASSR), 500, 100, 0, 2000, and 4000 HZ average auditory threshold is 80 dB. (C) The right ear air conduction click ABR threshold is 80 dBnHL. (D) The ABR threshold of 1 KHZ tone burst of right ear air conduction is 97 dBnHL. (E to F) Diagnostic distortion product otoacoustic emission (DPOAE) showed that meaningful DPOAE could not be induced at 750, 1000, 2000, 4000, and 8000 HZ. Sound transduction binaural “A” pattern, stapedius sound reflex cannot be extracted from the right ear, but can be extracted from the left ear. (G and H) MRI showed normal binaural structure.

**Figure 2.**
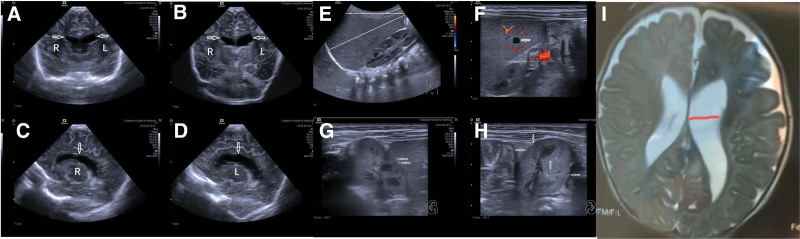
Imaging revealed other abnormalities in the proband. (A to D) Arrows showing bilateral lateral ventricle dilation, L: left, R: right. (E) The liver is enlarged, and the upper and lower diameter of the right liver (long white line) and the length under the right costal arch (short white line) both exceed the normal range. (F) The arrow shows multiple small points of strong echo in the right renal cortex: hamartoma. (G) Arrow shows splenic cyst. (H) Arrows indicate multiple small, punctate echoes in the left renal cortex: hamartoma. (I) MRI revealed widening of the lateral ventricles.

A 7-month-old girl (height 61.5 cm, weight 5.8 kg, both 3 percentile) was admitted to Changsha Hospital for Maternal & Child Health Care because of developmental retardation and low muscle tension. Head circumference (cm): 41, age at measurement: 0.5, stunted growth/short stature, special face, depression of the suprazygomatic muscles on both sides. The patient was a G2P2 patient with a weight of 2.6 kg at 38 weeks gestation. A 5-year-old girl with no developmental abnormalities. Apgar score is unknown. The mother had no special pregnancy, denied the history of intrauterine distress, and denied the history of anoxia and asphyxia during childbirth. The patient’s head is short in duration, the upright head is unstable, the auxiliary turn can be, can’t sit forward, does not actively grasp, can pronounce, the muscle tension is slightly low, and the head tilts to the right. Hearing screening failed right ear (Fig. 1). MRI showed slightly wider lateral ventricles on both sides(Fig. 2). After half a year of training, the child’s intelligence and language development were relatively normal, but unilateral deafness did not improve (Fig. 1), and new symptoms of liver enlargement and renal cortical hamartoma were found (Fig. 2A and B).

**Figure 3.**
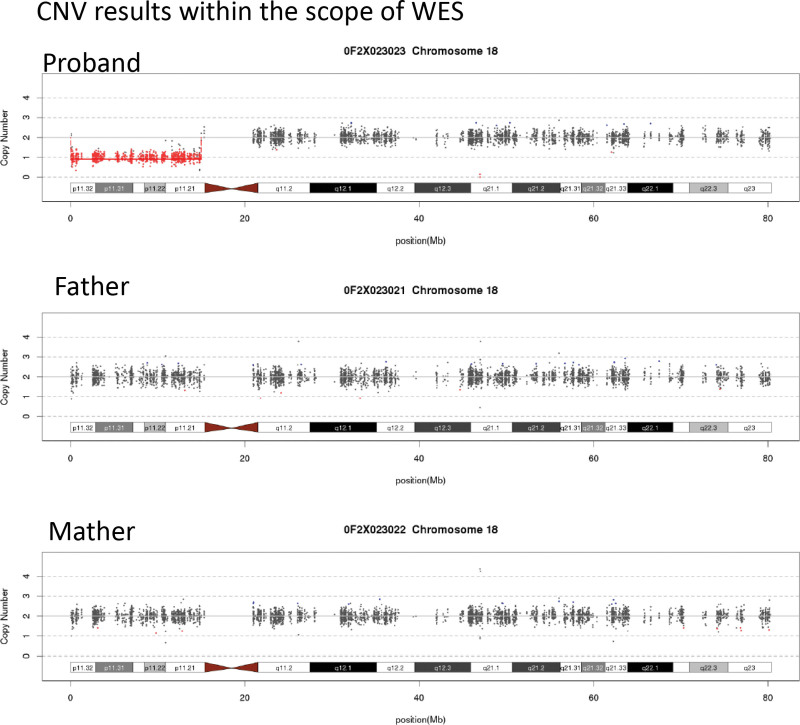
CNV results within the scope of whole-exome sequencing showed that the proband had A 14.95Mb region was found missing at p11.32-p11.21 on chromosome 18.

To further confirm this diagnosis at the molecular level, whole-exome sequencing and whole-genome copy number variation (CNV-seq) detection were performed on the genomic DNA of the patient and her parents. Data were analyzed by Berry Gene’s Verita Trekker variant site detection system and Enliven Variant Annotation Interpretation System. A 14.95Mb region was found missing at p11.32-p11.21 on chromosome 18 (FIG. 3), and the pathogenicity rating of this CNV was Pathogenic according to American College of Medical Genetics and Genomics (ACMG) guidelines. It involved 58 RefSeq protein-coding genes (1A,0; 3C,0.9 points): USP14; THOC1; COLEC12; CETN1; CLUL1; TYMS; ENOSF1; YES1; ADCYAP1; METTL4; NDC80; SMCHD1; EMILIN2; LPIN2; MYOM1; MYL12B; TGIF1; DLGAP1; AKAIN1; ZBTB14; EPB41L3; L3MBTL4; ARHGAP28; LAMA1; PTPRM; RAB12; GACAT2; MTCL1; NDUFV2; ANKRD12; TWSG1; RALBP1; PPP4R1; RAB31; TXNDC2; VAPA; APCDD1; NAPG; PIEZO2; GNAL; CHMP1B; MPPE1; IMPA2; CIDEA; TUBB6; AFG3L2; PRELID3A; SPIRE1; PSMG2; PTPN2; SEH1L; CEP192; LDLRAD4; FAM210A; RNMT; MC5R; MC2R; ANKRD30B.

It has been reported in the literature that the main clinical manifestations of a patient with 18p11.32-p11.31(chr18:148993–4748092,hg19) deletion were global developmental delay, epilepsy, special facial features, finger abnormalities, motor and language development delays.^[6]^ The main clinical manifestations of a patient with 18p11.32-p11.31(chr18:136226–4925310,hg19) deletion included forebrain acysmal deformity, congenital heart defect, special face, lip/palate cleft, etc.^[7]^ One patient with 18p11.32-p11.31 deletion was characterized by muscular dystrophy (FSHD).^[8]^ The main clinical manifestations of a 46,XY,del(18)(18)(p11.23)mat patient were acroschizencephaly, intrauterine growth retardation, special facial features, short stature, brain abnormalities, global growth retardation, diffuse hypotonic disorder, etc. The mother carried this missing fragment, and the main clinical manifestations were special facial features, intellectual impairment, short stature, etc.^[9]^ The main clinical manifestations of 1 patient with 18p11.32-p11.21 deletion were global growth delay, language delay, motor delay, and emotional and behavioral abnormalities.^[10]^

The new clinical symptoms in our case were unilateral deafness and hepatomegaly, deletion completely covered THOC1 gene [OMIM: 606930], and THOC1 mutation causes deafness, autosomal dominant 86 (type 86) [OMIM: 620280]. No case of a deletion in THOC1 has been reported to date. THOC1 gene was identified as haplodose insufficiency (HI) (gnomAD pLI score ≥ 0.9 and upper limit of o/e confidence interval < 0.35) by haplodose underdose (HI) ClinGen database prediction tool. DECIPHER pHaplo ≥ 0.86 (2H,0.15 points). Therefore, we determined that insufficient haploids of THOC1 caused unilateral deafness.

## 3. Discussion

The main clinical manifestations of 1 patient with 18p11.32-p11.21 deletion were global growth delay, language delay, motor delay, and emotional and behavioral abnormalities.^[10]^ But in this proband after 6 months of rehabilitation training speech, language and movement are back to normal. Therefore, the language and developmental delays caused by this deficiency can be recovered by subsequent rehabilitation training.

What is more interesting is that the progenitor developed a new clinical symptom of liver enlargement at the age of 1 year, which is helpful for subsequent studies to discover genes related to liver development in the missing genes. Recent studies show that the knockdown of THOC1 reduces the proliferation of hepatocellular carcinoma,^[11]^ and THOC1 is essential for mouse development,^[4]^ THOC1 may be the candidate gene for larger liver.

To date, no case of deletion in THOC1 has been reported, and our proposal cannot be confirmed by previous reports. Therefore, we hypothesized that the deletion of the THOC1 gene resulted in a haplodose of inadequate haplodose of modified gene expression, which ultimately led to unilateral deafness. This should be considered in the diagnosis of patients with THOC1 deletion in the future.

## 4. Methods

Genomic DNA was extracted from the peripheral blood of the patient and her parents. We performed whole-genome sequencing on the NovaSeq 6000 platform (Illumina, San Diego, USA) with a depth of 20X. The coverage of the proband, father and mother were 98.95%, 99.01%, and 98.87%, respectively. Raw image files were processed using CASAVA v1.82 for base calling and generating raw data. The sequencing reads were aligned to the human reference genome (hg38/GRCh38) using the Burrows–Wheeler Aligner tool, and PCR duplicates were removed by using Picard v1.57 (http://picard.sourceforge.net/). The Verita Trekker Variants Detection System by Berry Genomics and GATK software (https://software.broadinstitute.org/gatk/) were employed for variant calling. Variant annotation and interpretation were conducted by ANNOVAR (Wang, et al, 2010) and the Enliven Variant Annotation Interpretation System authorized by Berry Genomics. Annotation databases mainly include the following:

1. Human population databases, such as gnomAD (http://gnomad.broadinstitute.org/), the 1000 Genomes Project (https://www.internationalgenome.org/), the Berry big data population database, and dbSNP (http://www.ncbi.nlm.nih.gov/snp)
2. In silico prediction algorithms, such as SIFT (http://sift.jcvi.org), FATHMM (http://fathmm.biocompute.org.uk), Mutation Assessor (http://mutationassessor.org), CADD (http://cadd.gs.washington.edu), and SPIDEX (Xiong et al, Science 2015)
3. Disease and phenotype databases, such as OMIM (http://www.omim.org), ClinVar (http://www.ncbi.nlm.nih.gov/clinvar), HGMD (https://www.hgmd.cf.ac.uk/ac/index.php), and HPO (https://hpo.jax.org/)

The variants were classified into 5 categories–“pathogenic,” “likely pathogenic,” “uncertain significance,” “likely benign” and “benign”–according to the ACMG guidelines for interpretation of genetic variants (Richards et al, 2015). Variants with minor allele frequencies < 1% in the exonic region or with splicing impact were selected for further interpretation, considering the ACMG category, evidence of pathogenicity, and clinical synopsis and inheritance model of the associated disease.

For trio analysis, potential monogenetic inheritance patterns, including de novo, autosomal recessive, autosomal dominant, X-linked recessive inheritance and imprinted gene variation, were analyzed. Full penetrance was assumed for the potentially causal variants, and variants that were found in the parents or were recorded in any of the abovementioned databases or in our in-house control exomes were excluded as the etiology. Once a variant was considered to be the etiology of a recessive disorder, manual inspection for coverage and additional variants of the entire coding domain was undertaken using Integrated Genomics Viewer.

## Acknowledgments

We thank all of the patients and healthy volunteers who agreed to participate in the present study and all those who helped us successfully complete the research.

## Author contributions

**Data curation:** Xiangwen Peng, Geng Ouyang, Enhuan Yi.

**Funding acquisition:** Xiangwen Peng, Enhuan Yi, Xingxing Duan, Sifeng Wang.

**Writing – original draft:** Xiangwen Peng.

**Writing – review & editing:** Xiangwen Peng.

**Conceptualization:** Geng Ouyang, Enhuan Yi, Sifeng Wang.

**Formal analysis:** Enhuan Yi, Huali Qin, Xingxing Duan.

**Project administration:** Huali Qin.

**Resources:** Huali Qin.

**Methodology:** Xingxing Duan.
